# Supplementary material for: Early Serum Markers for Immune Checkpoint Inhibitor Induced Hypophysitis in Melanoma Patients
Source: Cancers (Basel). 2024 Mar 29;16(7):1340. doi: 10.3390/cancers16071340 (PMC11010823; doi:10.3390/cancers16071340)
Supplement: Supplementary file 1 [file cancers-16-01340-s001.zip › cancers-2880778-supplementary.pdf]

Supplemental Figure S1

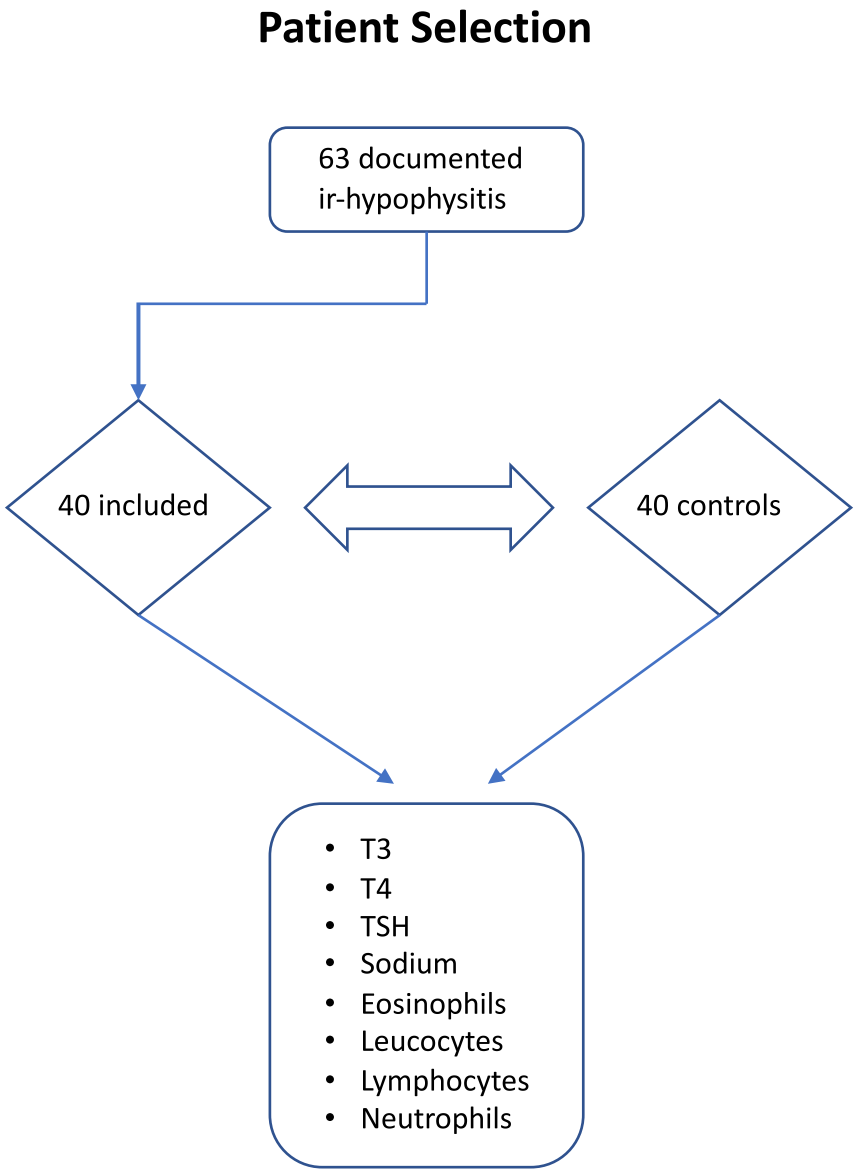

Supplemental Figure S1. The flow chart represents the study design

Supplemental Figure S2

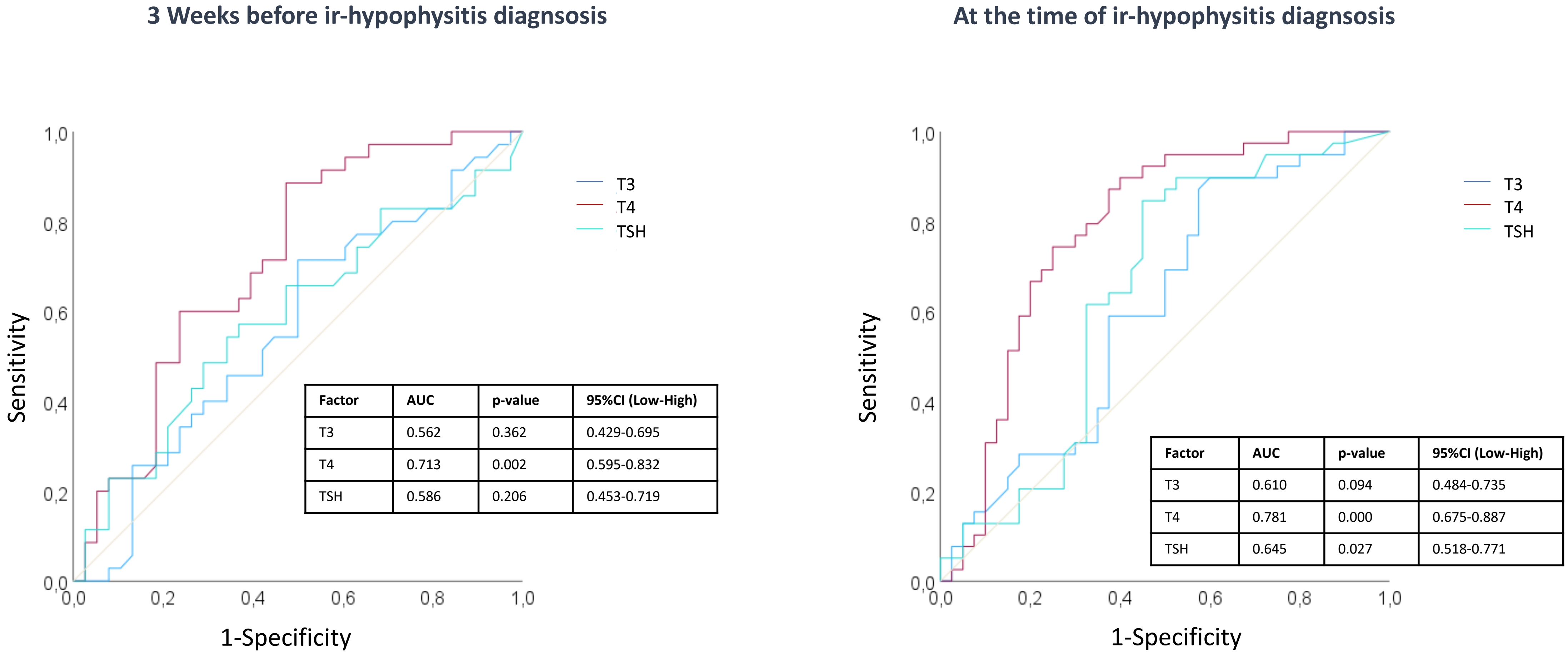

3 Weeks before ir-hypophysitis diagnsosis

Factor	AUC	p-value	95%CI (Low-High)
T3	0.562	0.362	0.429-0.695
T4	0.713	0.002	0.595-0.832
TSH	0.586	0.206	0.453-0.719

At the time of ir-hypophysitis diagnsosis

Factor	AUC	p-value	95%CI (Low-High)
T3	0.610	0.094	0.484-0.735
T4	0.781	0.000	0.675-0.887
TSH	0.645	0.027	0.518-0.771

Supplemental Figure S2. Comparision of ROC curves of T3 (blue), T4 (red), and TSH (green) at 3 weeks before ir-hypophysitis diagnosis (left) and at the time of ir-hypophysitis diagnosis (right) based on the development of hypophysitis. p-values, AUC and 95% CI for each marker are displayed on the table above the respective graph. p-value <0.05 is considered to be significant.

Supplemental Figure S3

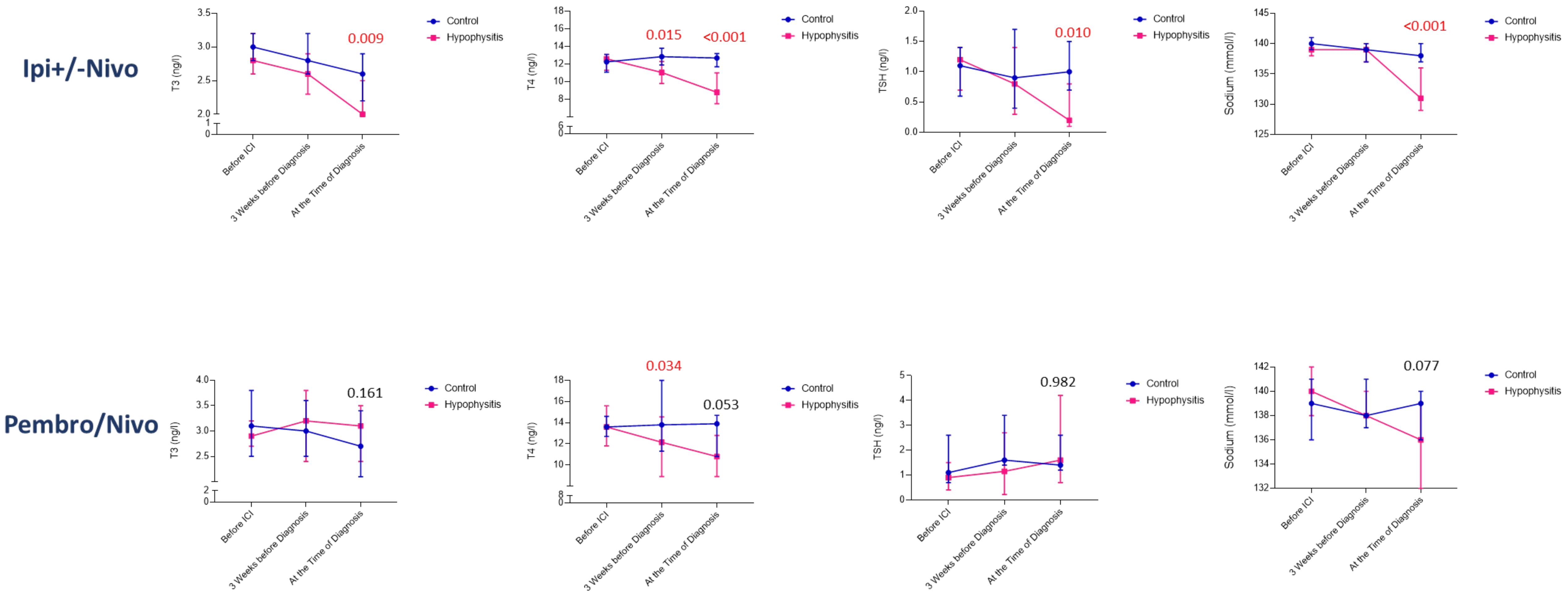

Supplemental Figure S3. Comparison of blood parameters at different time points between control (blue) and ir-hypophysitis patients (pink) in Ipi+/-Nivo and Pembro/Nivo treated patients. The lines represent the median and 95% CI for all charts, respectively. p-values are presented above the respective group on top of the chart, a p-value less than 0.05 indicates significant difference between the groups.

Supplemental Figure S4

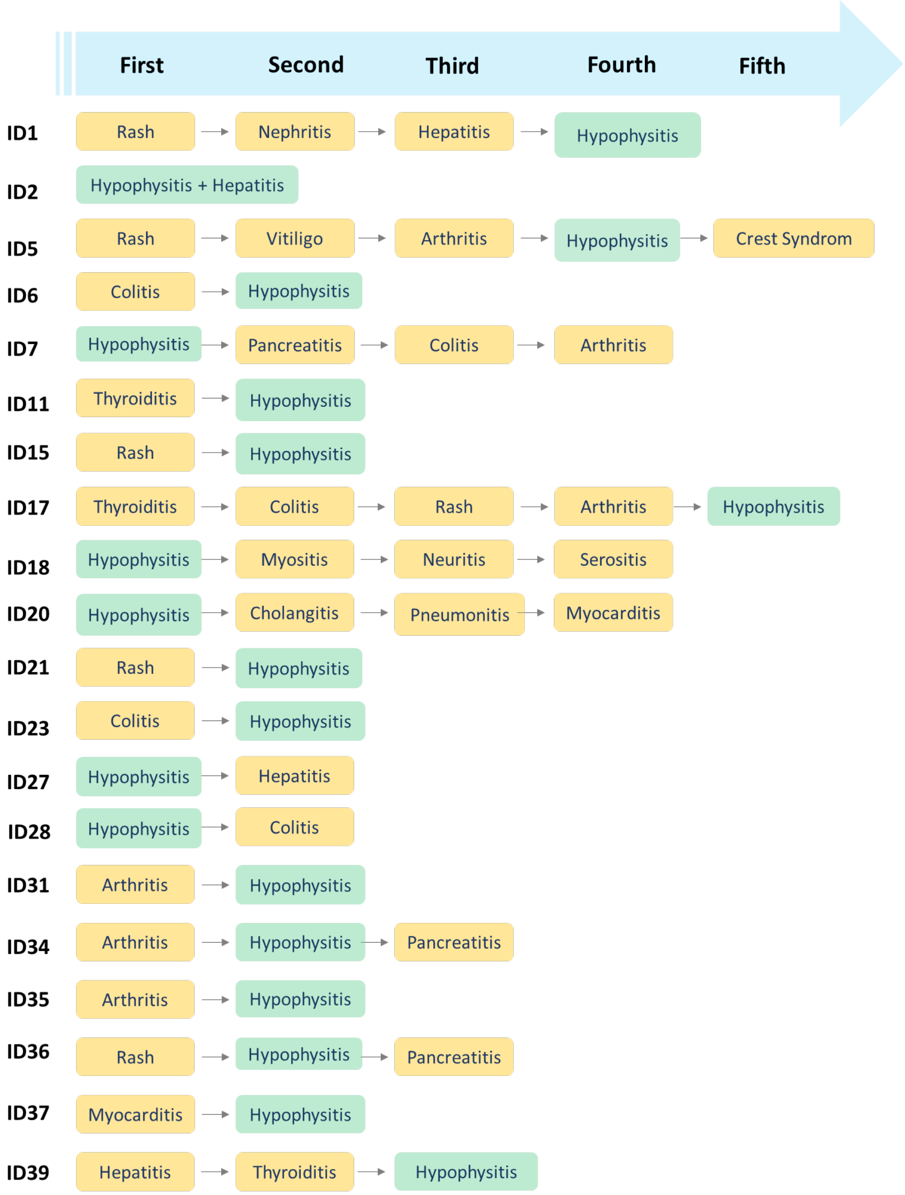

Supplemental Figure S4. Chronological order of other ir-AEs under ICI treatemnt in ir-hypophysitis group of patients
